# Diversity of Soil Filamentous Fungi Influenced by Marine Environment in São Luís, Maranhão, Brazil

**DOI:** 10.1155/2020/3727453

**Published:** 2020-05-01

**Authors:** Igor Vinícius Pimentel Rodrigues, Katia Regina Assunção Borges, Marcos Antonio Custódio Neto da Silva, Maria do Desterro Soares Brandão Nascimento, Juliano dos Santos, Alexandre Santana Azevedo, Geusa Felipa de Barros Bezerra

**Affiliations:** ^1^Programa de Pós-graduação em Saúde do Adulto, Núcleo de Imunologia Básica e Aplicada (NIBA), Universidade Federal do Maranhão, São Luís, Maranhão, Brazil; ^2^Programa de Pós-graduação em Clínica Médica, Universidade Estadual de Campinas (UNICAMP), Campinas, São Paulo, Brazil; ^3^Programa de Pós-graduação em Saúde do Adulto, Departamento de Patologia, Núcleo de Imunologia Básica e Aplicada (NIBA), Universidade Federal do Maranhão, São Luís, Maranhão, Brazil; ^4^Programa de Pós-graduação em Biodiversidade e Conservação, Universidade Federal do Maranhão, São Luís, Maranhão, Brazil; ^5^Bicho Nativo Consultoria, Universidade Federal do Maranhão, São Luís, Maranhão, Brazil

## Abstract

**Introduction:**

In recent decades, there has been an intensification of environmental problems, which are becoming increasingly critical and frequent due to population growth. Microorganisms, including soilborne fungi, play an essential role in maintaining and balancing the environment. One of the most impacted ecosystems in São Luís, Maranhão, Brazil, is the Jansen Lagoon State Park, an important tourist spot, which has suffered anthropogenic actions such as the dumping of household waste (sewage) in its body of water. As a consequence, these pollutants can accumulate in the adjacent soil, since the body of water is near this substrate. The objectives were to isolate and identify filamentous fungi from the soil of the Jansen Lagoon State Park.

**Methods:**

Monthly soil samples were collected and later processed using the modified suspension technique according to Clark (1965).

**Results:**

The isolated genera were *Aspergillus*, *Penicillium*, *Trichoderma*, *Absidia*, and *Fusarium. Aspergillus* is the fungal genus of greater dominance in the soil of the Jansen Lagoon State Park. *Aspergillus niger* was the dominant species (37%), followed by *A. tamarii* (21.6%).

**Conclusion:**

The main isolated fungi from the Jansen Lagoon State Park were *Aspergillus niger* and *Aspergillus tamrii.* These fungi can be used as biological markers of pollution and as biodegraders and/or bioremediators to improve the area studied.

## 1. Background

The twentieth century is a period in history marked by extremely dynamic civilizational and technological development, such as industrialization, wars, and intensive use and inappropriate disposal of heavy metals and synthetic xenobiotics, leading to many environmental problems [1]. Soil contamination is a problem that is becoming increasingly alarming in many countries mainly because of unbridled and often unplanned population growth. Contaminated lands pose a potential risk to human health, and the perception about this problem during the last few years has led to international remediation measures in these areas both in an attempt to alleviate the health risk arising from the side effects caused by the contamination of these environments and to make this resource reusable [2].

Currently, there are data generated by epidemiological studies showing the important role of fungi in respiratory diseases in the indoor and outdoor environments [3]. Fungal sensitization is not only found in patients with asthma, but it is now considered a risk factor for the development of asthma [4, 5].

However, fungi are also recognized for their remarkable ability to degrade complex natural materials, such as lignin, chitin, and cellulose, into simpler substances. Specifically, filamentous fungi are known for their distinct attributes to thrive under extreme pH, temperature, and nutrient variability conditions and to tolerate high metal concentrations [6–8]. Taking advantage of this, researchers have been using these microorganisms to remove pollutants from the environment. This process is called bioremediation and is ecologically correct and effective for the treatment of contaminated areas [2]. In Brazil, bioremediation still remains largely in the theoretical field, with few practical examples. However, there is a potential for expansion of this reality because Brazilian soil has physicochemical characteristics, such as high organic matter availability, that contribute to the degradation of several contaminants. In addition, environmental factors such as high temperature, relative humidity, and oxygen content create an environment conducive to the application of the bioremediation technique [6]. In São Luís, Maranhão, one of the most impacted ecosystems is the Jansen Lagoon State Park, which suffered from the impoundment of the stream that receives the same name, for the construction of Ana Jansen Avenue. This lagoon continuously receives releases of sewage whose toxic substances are deposited in the sediments and in the adjacent soil [9].

Therefore, this work aimed to isolate and identify filamentous fungi from the soil of the Jansen Lagoon State Park in São Luís do Maranhão.

## 2. Methods

### 2.1. Sample Collection

**Figure 1:**
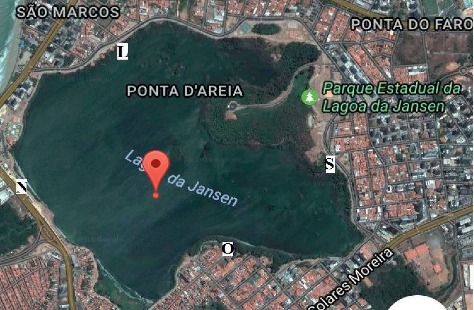
Study area location: Laguna da Jansen State Park and the four collection points: N, S, E, and W.

The soil was collected in the Jansen Lagoon State Park, which is located among São Francisco, Renascença I, Renascença II, Ponta D'Areia, and Ponta do Farol districts (Figure 1).

The geographic coordinates of the area are 2°29′56″S 44°17′59″W. It is located in the northwest region of São Luís, 4.0 km away from the Historic Center—World Heritage Site. Next to it are located the beaches of greater flow of bathers: Ponta D'Areia, Ponta do Farol, Marcela, and Calhau.

The Jansen Lagoon is a coastal body of water with characteristics of mangrove.

The water is brackish and rich in organic matter and allochthonous materials, i.e., its origin is not from the place where they are found. It occupies 140 hectares with an average depth of 1.0 m and the soil at the north (N) point of the Jansen Lagoon State Park is sandy and has a dark color due to the large amount of organic matter on it [10]. The surroundings presented a lesser flow of people, despite the fact that there is a little playground nearby.

The soil at the south point (S) is sandy and drier than that at the north point. It has an orange coloration and there is a large amount of litter on it. Plastic tailings were observed, probably due to the fact that it is located near a region where there is a greater flow of people (in front of a gym).

The soil at the east (L) point is located very near the body of water, being an extremely polluted region where it was observed direct discharge of sewage in the water through a pipe. The soil was very moist and muddy. Inadequately discarded plastic cups were observed on the site.

The soil at the west (O) point is also located near the body of water and is sandy, presented an orange coloration, and also there is a large amount of litter on it. It is a region with little flow of people.

Four (4) soil samples from the Jansen Lagoon State Park—São Luís, Maranhão, Brazil, were collected in the months of April, May, June, August, September, and October from each point: north (2°30′13″S 44°17′53″W), south (2°29′35″S 44°17′52″W), east (2°29′37″S 44°18′10″W), and west (geographic coordinate: 2°29′35″S 44°17′52″W).

The samples were obtained from a depth of up to 20 cm, using a sterile spoon according to da Silva et al. [11] with modifications because we did not randomize our samples. They were then placed in a zipped plastic bag and transported to the Laboratory of Mycology (NIBA/DEPAT/CCBS/UFMA) where they were processed.

The three first samplings occurred during the rainy season in São Luís, from April to June 2016. The first sampling occurred in April, and the average temperature was 30°C and the relative humidity was 75%. The second was carried out in May, where the average temperature was 32°C and the relative air humidity was 79%. The third was carried out in June, and the average temperature was 30°C and the relative humidity was 62% [12].

The three last samplings occurred in the dry season from August to October 2016. The fourth sampling was carried out in August, and the average temperature was 29°C and the relative humidity was 62%. The fifth sampling was performed in September, and the average temperature was 32°C and the relative humidity was 61%. The sixth sampling was performed in October, and the average temperature was 32°C and the relative humidity was 60% [12].

### 2.2. Isolation Method of the Filamentous Fungi Collected

To obtain the isolation of fungal colonies, the serial suspension technique was performed according to Clark [13]with modification, in which 25 g of soil sample was homogenized in 225 mL of sterile distilled water. The dilutions of 10^−2^ and 10^−3^ containing 0.1 mL of the suspension were pipetted on plates containing Sabouraud agar plus 10% chloramphenicol solution (100 mg/10 mL alcohol) [14]. Petri dishes were incubated at room temperature for five days until fungal colonies appeared (5–15 days according to the growth of fungi).

The different colonies were subcultured onto test tubes (18 × 180 mm) containing Sabouraud agar to work on fungal purification. Petri dishes containing specific culture media such as Czapek-Dox Agar and Potato Dextrose Agar (PDA) were also used for the identification of isolated fungi according to morphology and cultural features.

### 2.3. Identification of Filamentous Fungi Collected

The macroscopic characteristics (texture and coloration of the obverse and reverse of each colony) were observed. For the microscopic characteristics, the microculture technique was used according to Ridel [15] with modification, following the protocols already existing in the Mycology Laboratory (NIBA/DEPAT/CCBS/UFMA) [16–18] to ensure the visualization of the fruiting structures of each fungus. With this microscopic finding added to the macroscopic observations and, also, the aid of atlas [19], renowned scientific sites specialized in the taxonomy of microscopic fungi [20], and taxonomic keys [21], the taxonomy of the fungi was reached. A representative of each species was deposited in the Fungi Collection of the Federal University of Maranhão.

### 2.4. Data Analysis

The number of colony-forming units (CFU) was calculated by multiplying the number of isolated colonies by the inverse of the inoculated dilution, following the formula: CFU/mL = number of colonies/inverse of the dilution [22].

For the calculation of dominance of the species found in this study, the following formula was used [22]:

(1) $$\begin{array}{c} d=\frac{a_{i}}{\sum_{i=1}^{n}a_{i}}, \end{array}$$

where *d* is the dominance of the species; *a*_*i*_ is the total CFU of a given species; *n* and Σ*ai* are the sum of CFUs of all species.

For the calculation of species frequencies, the following formula was used [22]: *F* = *b*/*a* × 100, where *F* is the frequency of the species, *b* is the number of samples in which a given species was recorded, and *a* is the total number of samples.

### 2.5. Statistical Analysis

The data (number of CFUs isolated) were analyzed using the software Statistica version 7.0 for Windows (2007). The data of CFUs were transformed to log (*x*) in order to obtain a normal distribution. Rarefaction curve of samples was made in Biodiversity Pro Software [23]. Descriptive statistical techniques were used to assess all of the variables, with the aid of graphs and tables of frequencies. After that, the ANOVA test was performed followed by Tukey's posttest according to the analysis, with statistical significance if *p* ≤ 0.05.

## 3. Results

### 3.1. Dominance and Frequency of Fungal Species

Regarding fungi collection and identification, our results showed that *Aspergillus niger* was the dominant species (37%), followed by *A. tamarii* (21.6%), *Penicillium citrinum* (10%), *Aspergillus flavus* (8.7%), and *Trichoderma koningii* (7.7%).

*Aspergillus niger* was the most frequent species, found in 50% of all samples, *Aspergillus flavus* in 33%, *Aspergillus tamarii* and *Penicillium citrinum* in 17%, and *Aspergillus sclerotiorum* and *Aspergillus fumigatus* in 12% of the samples studied (Table 1).

### 3.2. Correlation of Environmental Factors and the Number of Colony-Forming Units Recovered

**Figure 2:**
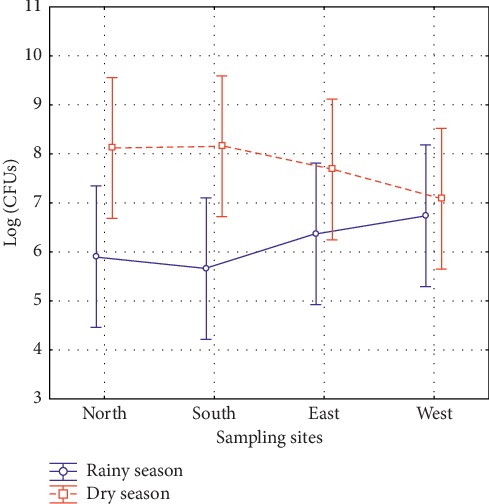
Frequency distribution of colony-forming units of fungi isolated in the rainy season (April to June 2016) and dry season (August to October 2016) in Jansen Lagoon State Park, São Luis, State of Maranhão.

Table 2 shows that we recovered a greater number of colony-forming units (CFUs) in the dry season (August to October) when compared to the rainy season (April to June) (*p*=0.0431). However, when the variables *sampling sites* and *seasons* were correlated, there was no statistically significant difference between them (*p*=0.41), as shown in Figure 2.

### 3.3. Rarefaction Curve of Fungal Species Collected

**Figure 3:**
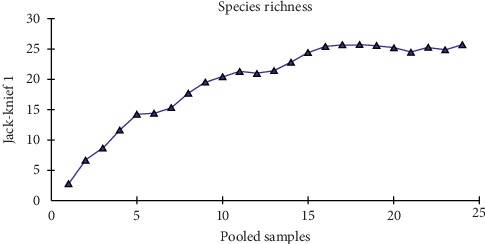
Rarefaction curve of the collected samples in Jansen Lagoon State Park, São Luis, State of Maranhão, in 2016.

The rarefaction curve showed that we made a satisfying sampling effort in this study, since the curve that represents the diversity of species collected stabilized at the end of the samplings, as shown in Figure 3.

## 4. Discussion

In a Brazilian Cerrado region, located in Mato Grosso, Garcia et al. found that *Penicillium* and *Aspergillus* were amongst the dominant fungal genera isolated, with percentages of 28% and 5%, respectively, which was reasonable since both the areas studied in this research and in our work are characterized by having a tropical climate with two distinct seasons: a dry and a wet season [24]. In this work, we found that *Penicillium* and *Aspergillus* were the dominant fungal genera, which was corroborated in other research studies using soil as the substrate [25], since we found twelve species belonging to the genus *Aspergillus* and three to the genus *Penicillium*.

During the dry season, greater fungal genera and colony-forming units were found, suggesting that, in this period, there is, probably, a greater dispersion of the propagules due to the greater intensity of the wind [26, 27] and a high concentration of spores in the air results in increases in allergic diseases of the respiratory system [28].

Among the fungi found in the dry season, we highlight two species that, according to the literature [29, 30], produce allergens substances that can trigger IgE-mediated allergic reactions: *Aspergillus fumigatus* and *Penicillium citrinum*. It is important to emphasize that these species were found in regions with a significant flow of people (south and east), which makes this an alarming public health issue.

The high fungal abundance found in both rainy and dry seasons can be explained by the characteristic environmental conditions of these climates. A high relative humidity is essential for the development of fungi, whereas sunny weather contributes to the release of spores [17].

It was demonstrated that the fungus *Aspergillus niger*, also isolated from contaminated soil, was able to remediate synthetic and industrial wastewaters after a biological system called Fenton treatment was utilized. It was observed that when this treatment and the microorganism were combined, the decolorization of the effluents was even more effective [31].

Also, another study found that two strains belonging to the *Penicillium* genera were able to degrade crude oil from a petroleum-polluted area, thus presenting a high activity in aliphatic hydrocarbon degradation [32]. A review pointed out that several *Trichoderma* species, including *T. koningii*, which was isolated in this work, possess the ability to metabolize a variety of both high- and low-molecular-weight polycyclic aromatic hydrocarbons (PAHs) such as naphthalene, phenanthrene, chrysene, pyrene, and benzo[a]pyrene. Therefore, these fungi present remarkable potential as remediation agents in polluted soils [7, 33].

A study reported the ability of a species belonging to the *Absidia* genus to decolorize Cresol Red 65%, a synthetic dye frequently used for monitoring the pH in aquaria, within 30 days under agitation condition [34]. All the fungal genera mentioned above were found in the present work, demonstrating that these fungi are able to adapt to environments with some degree of pollution and can be used as bioindicators of contamination.

A research carried out in São Luís, Maranhão, Brazil, revealed that *Aspergillus flavus* was able to biodegrade polypropylene, which is a polymer derived from recyclable plastic, while *Aspergillus terreus* showed potential to degrade polyethylene terephthalate, also a thermoplastic polymer used as fibers for weaving and for packaging of beverages [35]. The fungi used in this research were isolated from the soil of Aterro da Ribeira, São Luís, Maranhão, Brazil, and retrieved from the Fungi Collection of the Federal University of Maranhão. These two fungal species were also found in the area studied in this research, where the presence of inadequately dumped plastic materials was visible, indicating that these species, if arranged in a microbial consortium, can contribute to the remediation of this environment.

We notify that *Aspergillus* is the dominant fungal genus in the soil of Jansen Lagoon State Park; *Aspergillus niger* and *Aspergillus tamarii* are the predominant fungi in the soil of Jansen Lagoon State Park. The presence of the fungi found in this study serves as a guide for future bioremediation actions in this studied area, which are urgent, since the Jansen Lagoon State Park is an important tourist point of São Luís and the pollution found in this ecosystem represents a waste of its tourism potential and poses a health risk for the people who attend this place.

All the fungi isolated in this work are stored in the Fungi Collection of the Federal University of Maranhão and available to researchers of the area. Each isolated fungus receives an identifying number to facilitate posterior use. The fungi collection is available to the public in general.

## Figures and Tables

**Table 1:** Total number of colony-forming units of fungi isolated in Jansen Lagoon State Park, São Luis, State of Maranhão, between April and October 2016, per sampling sites.

Fungus	North (CFU/mL)		South (CFU/mL)		East (CFU/mL)		West (CFU/mL)		Total (CFUs)	
	*n*	%	*n*	%	*n*	%	*n*	%	*n*	%
*Aspergillus flavus*	3.10^2^	1.64	11.10^2^	10.28	27.10^2^	21.09	5.10^2^	4.47	46.10^2^	8.71
*Aspergillus sclerotiorum*	2.10^2^	1.1	1.10^2^	0.94	0	0	0	0	3.10^2^	0.57
*Aspergillus flavipes*	3.10^2^	1.64	0	0	0	0	0	0	3.10^2^	0.57
*Aspergillus candidus*	10.10^2^	5.5	2.10^2^	1.86	0	0	0	0	12.10^2^	2.27
*Aspergillus tamarii*	106.10^2^	58.24	3.10^2^	2.8	1.10^2^	0.78	4.10^2^	3.53	114.10^2^	21.6
*Aspergillus avenaceus*	0	0	0	0	0	0	3.10^2^	2.65	3.10^2^	0.57
*Aspergillus niger*	25.10^2^	13.73	61.10^2^	57	55.10^2^	42.97	55.10^2^	48.67	196.10^2^	37.12
*Aspergillus terreus*	0	0	1.10^2^	0.94	10.10^2^	7.82	1.10^2^	0.88	12.10^2^	2.27
*Aspergillus oryzae*	0	0	3.10^2^	2.8	0	0	3.10^2^	2.65	4.10^2^	1.04
*Aspergillus fumigatus*	0	0	10.10^2^	9.35	2.10^2^	1.56	0	0	12.10^2^	2.27
*Aspergillus alliaceus*	1.10^2^	0.55	0	0	0	0	0	0	1.10^2^	0.19
*Aspergillus ochraceus*	0	0	4.10^2^	3.74	0	0	1.10^2^	0.88	5.10^2^	0.94
*Penicillium decumbens*	1.10^2^	0, 55	0	0	0	0	0	0	1.10^2^	0.19
*Penicillium citrinum*	20.10^2^	11	10.10^2^	9.35	23.10^2^	17.96	0	0	53.10^2^	10
*Penicillium purpurogenum*	10.10^2^	5.5	0	0	0	0	0	0	10.10^2^	1.9
*Absidia corymbifera*	0	0	0	0	10.10^2^	7.82	1.10^2^	0.88	11.10^2^	1.9
*Fusarium solani*	0	0	1.10^2^	0.94	0	0	0	0	1.10^2^	0.19
*Trichoderma koningii*	1.10^2^	0.55	0	0	0	0	40.10^2^	35.39	41.10^2^	7.7

Total	182.10^2^	100.0	107.10^2^	100.0	128.10^2^	100.0	113.10^2^	100.0	528.10^2^	100.0

CFUs: colony-forming units.

**Table 2:** Total averages of CFUs per sampling sites and per season, in Jansen Lagoon State Park, São Luis, State of Maranhão, in 2016.

	North	South	East	West	Total average of CFUs/mL per season
Rainy season	500 UFC/mL	300 UFC/mL	1533.33 UFC/mL	1900 UFC/mL	1058.33^A^
Dry season	5233.33 UFC/mL	3600 UFC/mL	2733.33 UFC/mL	1933.33 UFC/mL	3374.99^B^
Total average of CFUs per sampling sites	2866.66 UFC/mL	1950 UFC/mL	2133.22 UFC/mL	1916.66 UFC/mL	

^A,B^Significantly different averages by Tukey's test (*α* = 0.05).

## Data Availability

The data of this research are all included in the manuscript and the authors have the data bank with them.
